# CDKN2B-AS1 Promotes Malignancy as a Novel Prognosis-Related Molecular Marker in the Endometrial Cancer Immune Microenvironment

**DOI:** 10.3389/fcell.2021.721676

**Published:** 2021-10-12

**Authors:** Di Yang, Jian Ma, Xiao-Xin Ma

**Affiliations:** ^1^Department of Gynecology, Cancer Hospital of China Medical University, Liaoning Cancer Hospital and Institute, Shenyang, China; ^2^Department of Obstetrics and Gynecology, Shengjing Hospital of China Medical University, Shenyang, China

**Keywords:** endometrial cancer, lncRNA, prognostic signature, immune infiltration, CDKN2B-AS1

## Abstract

The prognosis of patients with endometrial cancer (EC) is closely associated with immune cell infiltration. Although abnormal long non-coding RNA (lncRNA) expression is also linked to poor prognosis in patients with EC, the function and action mechanism of immune infiltration-related lncRNAs underlying the occurrence and development of EC remains unclear. In this study, we analyzed lncRNA expression using The Cancer Genome Atlas and clinical data and identified six lncRNAs as prognostic markers for EC, all of which are associated with the infiltration of immune cell subtypes, as illustrated by ImmLnc database and ssGSEA analysis. Real-time quantitative polymerase chain reaction showed that CDKN2B-AS1 was significantly overexpressed in EC, whereas its knockdown inhibited the proliferation and invasion of EC cells and the *in vivo* growth of transplanted tumors in nude mice. Finally, we constructed a competing endogenous RNA regulatory network and conducted Gene Ontology enrichment analysis to elucidate the potential molecular mechanism underlying CDKN2B-AS1 function. Overall, we identified molecular targets associated with immune infiltration and prognosis and provide new insights into the development of molecular therapies and treatment strategies against EC.

## Introduction

Endometrial cancer (EC) is one of the most common malignant tumors of the female reproductive system, and its incidence has increased in recent years, particularly in younger females (Smrz et al., 2020). In 2020, approximately 65,620 new cases of EC in the United States and 12,590 related deaths have been reported, with a mortality rate second only to ovarian cancer (Siegel et al., 2020). The efficacy of the currently available treatments for patients with recurrent or advanced EC remains poor (Leslie et al., 2021); however, novel immunotherapies have attracted significant attention as potential treatment strategies for patients with EC (Meng et al., 2021). It is therefore important to explore and develop reliable immune-related targets and prognostic markers for EC for individualized treatment of EC.

In 2013, The Cancer Genome Atlas (TCGA) had proposed a molecular typing system that divided EC into four subtypes, namely, DNA polymerase epsilon (POLE), microsatellite instability (MSI), copy number abnormalities low (CNL), and copy number abnormalities high (CNH) (Du et al., 2019). These subtypes can help to more accurately classify post-operative treatments (including immunotherapy) and prognosis of patients with EC than the traditional pathomorphology (Zhou et al., 2021). In POLE hypermutant and MSI EC, PD-1, and PD-L1 expression are correlated with the degree of T lymphocyte infiltration, with a higher number of infiltrating T lymphocytes eliciting a stronger local immune response associated with better prognosis (Liu et al., 2021). The diagnosis and treatment of EC has influenced its molecular classification (Kahn et al., 2019), and this has prompted the use of immunotherapies based on the tumor microenvironment (TME), genotype, and epigenetic alterations. Although some patients with EC have shown encouraging results following immunotherapy, some have failed to respond (Meng et al., 2021). However, the therapeutic efficacy of immune checkpoint inhibitors can be predicted based on PD-L1 expression, mutational load, lymphocyte infiltration, and mutation-related neoantigens (Post et al., 2020).

Less than 2% of the human genome encodes transcribable protein-coding genes, whereas 85% encodes non-coding RNAs. Long non-coding RNAs (lncRNAs) are RNAs over 200 nucleotides in length that do not encode proteins but play key roles in regulating transcription and post-transcriptional events affecting cell function (Zheng et al., 2019). LncRNA are being actively explored as biomarkers, supporting their prevalent link with diseases. For example, through experiments in humans and mice showed that there is an lncRNA locus maenli on human chromosome 2 (Allou et al., 2021), and its deletion result in human Mendelian disease. Transcription of the lncRNA upperhand controls Hand2 expression and heart development (Anderson et al., 2016). Such promising developments suggest that the entrance of lncRNA-based therapeutics into clinical testing is imminent. Recent findings have demonstrated that lncRNAs can shape the TME by changing the internal characteristics of tumor cells, thereby promoting and maintaining tumor occurrence and development (Dong et al., 2019). In breast and ovarian cancer cells, the lncRNA Xist mediates macrophage polarization by competing with microRNA (miR)-101 to regulate KLF6 expression, thereby affecting proliferation and migration (Zhao et al., 2021). Similarly, the lncRNA HEIH can improve the immunogenicity of triple-negative breast cancer (TNBC) cells and regulate TNBC progression by inducing MICA/B and suppressing the immune checkpoint inhibitor, PD-L1 (Nafea et al., 2021). LncRNAs can induce tumor occurrence and metastasis by activating the immune system and responses, including antigen release, antigen presentation, immune cell differentiation, immune cell migration, and T cell infiltration (Huang et al., 2018). However, the roles of immune-related lncRNAs in EC remain to be investigated.

In this study, we analyzed results for EC samples in TCGA and identified six lncRNAs, namely, PRRT3-AS1, LINC01503, CDKN2B-AS1, LINC01629, LINC01833, and LINC01936, associated with the immune microenvironment whose high expression may be correlated with poor patient prognosis. We also analyzed the correlation between the expression of these lncRNAs and immune cell infiltration, common immune checkpoints, MHC molecules, chemokines, and chemokine receptors. In addition, their expression was verified using real-time polymerase chain reaction (PCR) in EC tissues and normal tissues while *in vitro* and *in vivo* experiments confirmed that CDKN2B-AS1 knockdown inhibited EC cell proliferation and invasion, as well as their ability to form tumors. Together, the findings of this study provide new insights and theoretical basis for future immunotherapies to treat EC.

## Materials and Methods

### Identification of Immune-Related Long Non-coding RNAs in Endometrial Cancer

The transcriptome profiles and corresponding clinical data of 575 patients with EC were downloaded from TCGA, including 552 patients with UCEC and 23 normal samples. First, we screened differentially expressed genes (DEGs) using the “limma” program package with the following criteria: | log2FC| > 1 and FsDR < 0.05. A heat map and volcano map were produced from the resulting DEGs. Immune-related lncRNA genes were obtained using the official ImmLnc website. Differentially expressed immune-related lncRNAs were screened using | log2FC| > 1 and *p* < 0.05 and then analyzed using the Edger software package.

### Identification of Immune-Related Long Non-coding RNA Prognostic Indicators for Endometrial Cancer

To screen immune-related lncRNAs associated with survival from the clinical EC data from TCGA, we performed univariate Cox proportional hazard regression (PHR) analysis with *p* < 0.001. Multivariate Cox PHR analysis was used to identify prognostic markers, while the risk score of each patient was calculated based on lncRNA expression levels. According to the median risk score, patients with EC were divided into high- and low-risk groups. Univariate and multivariate Cox regression analyses were then used to evaluate the relationships between risk score and age, tumor grade, tumor differentiation, and depth of invasion. Survival differences between the two groups were determined using Kaplan–Meier (KM) survival analysis.

### Correlation Analysis of Immune Cell Infiltration

Immune infiltration analysis was performed based on the ssGSEA scores of activated dendritic cells (aDCs), B cells, CD8 T cells, cytotoxic cells, DCs, eosinophils, immature DCs (iDCs), macrophages, mast cells, neutrophils, natural killer (NK) CD56bright cells, NK CD56dim cells, NK cells, plasmacytoid DCs (pDCs), T cells, T helper cells, T central memory cells (Tcm), T effector memory cells (Tem), T follicular helper (Tfh), T gamma delta (Tgd), Th1 cells, Th17 cells, Th2 cells, and Treg cells to determine whether immune infiltration correlated with lncRNA expression. The online analysis website, ImmLnc,^1^ was used to determine the immune-related functions of lncRNAs in cancer, as well as lncRNA pathways, correlations between lncRNAs and immune cell types, and cancer-related lncRNAs.

### Construction of the ceRNA Regulatory Network

Differentially expressed lncRNAs and miRNAs were paired using the miRcode database. Based on the miRDB, miRTarBase, and TargetScan databases, starBase online software was used to predict the target genes of the screened differentially expressed miRNAs to obtain a ceRNA regulatory network diagram of lncRNA–miRNA–mRNAs using the visual analysis software, Cytoscape V3.5.2.

### Functional Enrichment Analysis

We used Metascape^2^ to analyze the pathways enriched by the 100 molecules most relevant to CDKN2B-AS1.

### Patients and Samples

This study enrolled patients (aged 23–69-years-old) who had undergone surgical uterus removal at the Department of Obstetrics and Gynecology, Shengjing Hospital Affiliated to China Medical University, from 2017 to 2018. We collected 20 EC tissue samples and 20 normal endometrial tissue samples. No patients had received anti-cancer treatments, such as radiotherapy, chemotherapy, or immunotherapy, prior to surgery. Histopathological analysis was performed by two pathologists. The study was approved by the Ethics Committee of Shengjing Hospital Affiliated to China Medical University (Ethics number: 2018PS251K). All study participants provided informed consent.

### Cell Culture

Ishikawa cells were cultured in RPMI 1640 medium (Gibco, Carlsbad, CA, United States), while HEC-1A cells were cultured in McCoy’s 5A medium (Gibco). Both cell lines were obtained from the Institute of Biochemistry and Cell Biology at the Chinese Academy of Sciences (Shanghai, China).

### Fluorescence *in situ* Hybridization

To determine the localization of lncRNAs in EC cells, fluorescence *in situ* hybridization (FISH) probes (RiboBio, Guangzhou, China) were constructed. Both Ishikawa and HEC-1A cells were treated with 1% paraformaldehyde at 37°C for 1 h and then incubated with the hybridization probe at 37°C for 16 h. On the second day, the cells were washed with hybridization solutions I, II, and III, and images were taken and analyzed using a fluorescent inverted microscope (20× magnification).

### Cell Transfection

CDKN2B-AS1 interference lentiviral vectors and a corresponding negative control (NC) were purchased from GenePharma (Shanghai, China) and transfected at a multiplicity of infection (MOI) of 50. Cells were transfected using Lipofectamine 3000 (Invitrogen) according to the manufacturer’s instructions, and stably transfected cells were selected using puromycin. The primers used for cloning and the shRNA sequences are listed in Supplementary Table 1.

### Cell Proliferation Assay

During their logarithmic growth phase, Ishikawa and HEC-1A cells were transferred into 96-well plates that included a blank control and three parallel wells per group. Cell proliferation was detected using an EdU cell proliferation detection kit (RiboBio, Guangzhou, China) according to the manufacturer’s instructions. Briefly, cells were incubated with 50 μM of pre-prepared EdU mixed reagent for 2 h, fixed, and subjected to DNA staining. The cells were then washed with phosphate-buffered saline (PBS) and images were acquired and analyzed using a fluorescence microscope (Nikon, Japan) at 20× magnification.

### Cell Invasion Assay

Sterilized Transwell chambers were coated with Matrigel (pore size 8 μM; Corning, NY, United States) and incubated at 37°C overnight to solidify. Logarithmic phase cells were harvested, centrifuged, and resuspended in serum-free medium after the supernatant had been discarded. Next, 800 μL of culture medium (containing 10% fetal bovine serum) was added to a 24-well plate and the Transwell chamber was added. The cell suspension (5 × 10^4^ cells) was then added to the upper chamber and incubated for 24 h at 5% CO_2_ and 37°C. After the Transwell chamber had been removed, the cells in each well were absorbed and the liquid in the upper chamber was discarded. The wells were then air-dried, fixed with 4% paraformaldehyde, stained with crystal violet dye, and rinsed with PBS. Images were acquired under a microscope (Nikon, Japan) and the cells were counted.

### RNA Extraction and Quantitative Real-Time-Polymerase Chain Reaction

Total RNA was extracted from cultured cells or tissues using TRIzol reagent (Takara, Shiga, Japan) and reverse-transcribed into cDNA using a PrimeScript^*TM*^ RT-PCR Kit (Takara) with SYBR^®^ TB Green^*TM*^ Premix Ex Taq II (Takara). PCR-specific primers were designed by Sangon Biotech (Shanghai, China). Fold changes in expression were calculated using the 2^−^ΔΔ^Ct^ method, with *GAPDH* as an internal control. The primer sequences used are listed in Supplementary Table 1.

### Tumor Xenografts in Nude Mice

All animal experiments were approved by the Ethics Committee of Shengjing Hospital Affiliated to China Medical University and were conducted in strict accordance with ethical standards. Athymic BALB/c nude mice (4–6 weeks old) were purchased from HFK Bioscience (Beijing, China). For the *in vivo* study, CDKN2B-AS1 shRNA and control shRNA stably transfected Ishikawa cells were harvested, the underarm tumor of each mouse was injected with transfected cells suspended in 10^6^/100 μL PBS, tumor sizes were measured by caliper and recorded every 4 days. The mice were sacrificed after 28 days. Tumors were excised, placed in 4% paraformaldehyde, and embedded in paraffin.

### Statistical Analysis

Statistical analyses were performed using R software (Version3.5.1) and GraphPad Prism 8 software (GraphPad, La Jolla, CA, United States). Differences between count data were calculated using the χ^2^ test, with the data expressed as the mean ± SEM. Data between two groups were compared using unpaired *t*-tests. *p*-Values of <0.05 were considered statistically significant.

## Results

### Analysis of Differentially Expressed Immune-Related Long Non-coding RNAs in Endometrial Cancer Tissues

**FIGURE 1:**
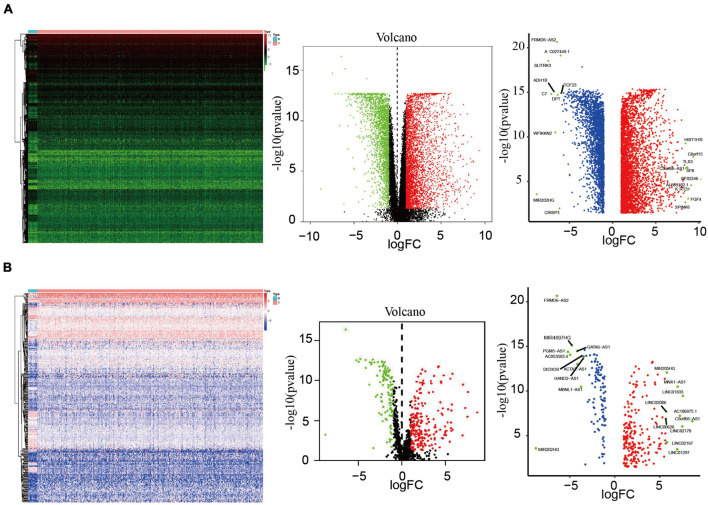
Analysis of differentially expressed lncRNAs. **(A)** Heatmap and volcano map of 552 EC samples and 23 normal endometrium samples showing 3,860 upregulated molecules and 2,406 downregulated molecules (fold change >4, *p* = 0.001), and showed the most differentially expressed molecules. **(B)** Heatmap and volcano plots of 340 differentially expressed lncRNAs showing 228 upregulated and 112 downregulated, and showed the most differentially expressed molecules.

We analyzed the data of EC samples (*n* = 552) and normal endometrium samples (*n* = 23) in the TCGA database, with criteria of | log2FC| > 1 and FsDR < 0.05, and identified a total of 6,266 DEGs, of which 3,860 were upregulated and 2,406 were downregulated (Figure 1A). Immune-related lncRNA gene information was obtained using the ImmLnc website and 340 differentially expressed immune-related lncRNAs were screened according to the nature of the gene, of which 228 were upregulated and 112 were downregulated (Figure 1B).

### Identification and Prognostic Characteristics of Six Immune-Related Long Non-coding RNAs

**FIGURE 2:**
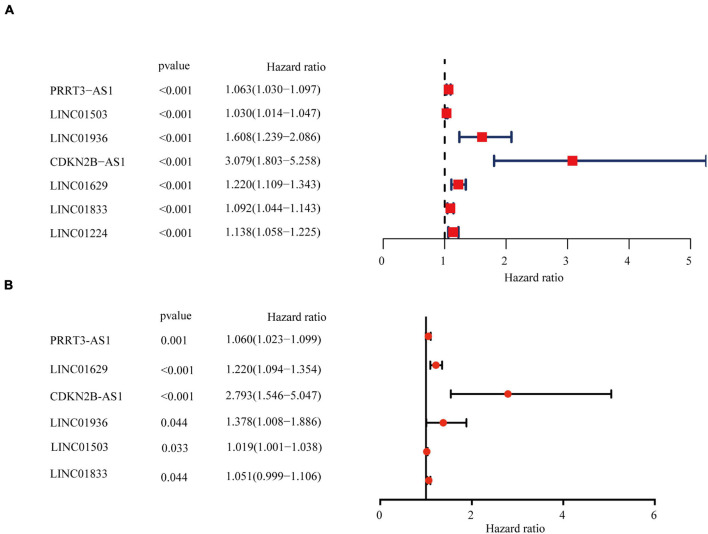
Identification and assessment of the immune-related lncRNA prognostic signature for EC. HR and *p*-values from univariate Cox **(A)** and multivariate Cox **(B)** HR regression of selected genes and immune terms (criteria: *p*–value < 0.001).

To predict the prognosis of EC patients, we constructed a risk scoring model based on the survival data for the EC samples. Univariate Cox PHR analysis on the expression profiles of 340 lncRNAs identified seven immune-related lncRNAs with *p* < 0.001 whose expression was related to prognosis (Figure 2A). Subsequent stepwise multiple Cox regression analysis determined that the expression of six immune-related lncRNAs was related to prognosis: PRRT3-AS1, LINC01503, LINC01936, CDKN2B-AS1, LINC01629, and LINC01833 (Figure 2B). The coefficient of each molecule factors were calculated with multivariate Cox proportional risk regression analysis. The risk score was then calculated for each sample based on the expression levels of these six lncRNAs using the following equation (Table 1):


Riskscore=(0.06×PRRT3)-AS1+(0.02×LINC01503)+(0.32+LINC01936)+(1.03+CDKN2B)-AS1+(0.20+LINC01629)+(0.05×LINC01833).

**TABLE 1:** The expression levels of these six lncRNAs.

**ID**	**PRRT3-AS1**	**LINC01503**	**LINC01936**	**CDKN2B-AS1**	**LINC01629**	**LINC01833**
Lower limit	1.023583	1.00155	1.008181	1.545618	1.09442	0.999163
Relative risk	1.060732	1.019693	1.378964	2.793029	1.217429	1.05138
Upper limit	1.09923	1.038164	1.886111	5.04718	1.354264	1.106327
*p*-Value	0.001189	0.033254	0.04433	0.000668	0.000294	0.043889
Coefficient	0.058959	0.019501	0.321332	1.027127	0.196742	0.050104

### Correlation Between the Immune-Related Long Non-coding RNA Signature and the Overall Survival and Prognosis of Patients With Endometrial Cancer

**FIGURE 3:**
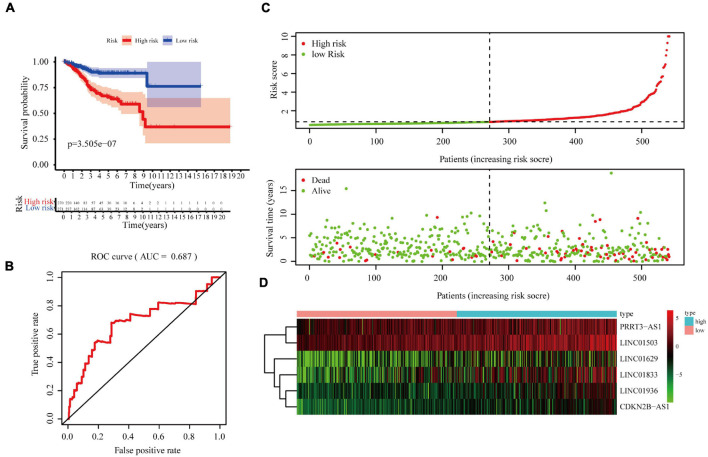
Correlation between the six immune-related lncRNA signature and the OS and prognosis of patients with EC. **(A)** Kaplan–Meier OS curves for the high- and low-risk groups. **(B)** Risk curve of each sample reordered by risk score. **(C)** Scatter plot of OS. Green and red dots represent survival and death, respectively. **(D)** Heatmap showing the expression profiles of the signature in the low- and high-risk groups. The pink and blue bars represent the low-and high-risk groups, respectively.

According to the median risk score, EC samples were divided into high- and low-risk groups and a KM curve was created to calculate overall survival (OS) rates. The high-risk group displayed poor OS, indicating that the risk score effectively evaluated prognosis [*p* = 3.505e^–07^, area under the curve (AUC) = 0.687; Figures 3A,B]. The risk curve and scatter plot were generated to show the risk score and survival status of each endometrial cancer sample. The risk coefficient and mortality of samples in the high-risk group were higher than those in the low-risk group (Figure 3C). A heat map of the expression profiles of the six lncRNAs in EC samples showed that all six were highly expressed in the high-risk group (Figure 3D). Together, these findings suggest that the six immune-related lncRNAs can be used as prognostic markers for EC.

To verify the applicability of this prognostic signature based on the complete TCGA data set, we randomly divided 541 patients with EC in TCGA into two test sets (1, *n* = 271, Supplementary Figure 1, 2, *n* = 270, Supplementary Figure 2) to build a verification model. Consistent with the results observed in the entire data set, the OS rate of patients in the high-risk group was lower than that of patients in the low-risk group in both test sets 1 and 2 (Supplementary Figure 1A, *p* = 4.059e^–06^ and Supplementary Figure 2A, *p* = 4.034e^–03^, respectively). In addition, the receiver operating characteristic (ROC) curve showed good performance, with AUCs for 5-year OS of 0.711 (Supplementary Figure 1B) and 0.675 (Supplementary Figure 2B).

### Relationship Between the Immune-Related Long Non-coding RNA Signature and Clinicopathological Parameters of Endometrial Cancer

**FIGURE 4:**
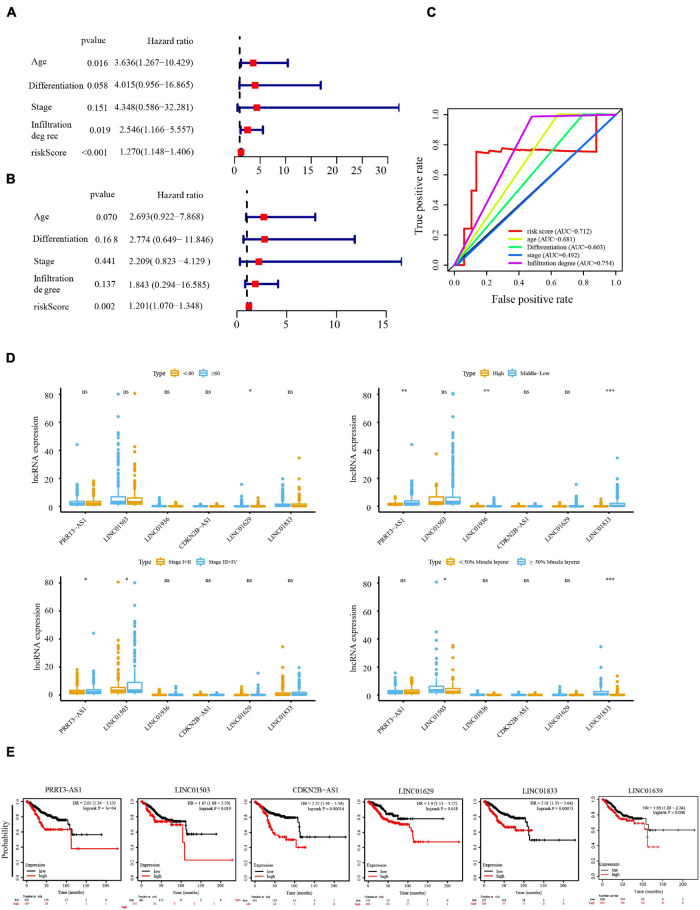
Cox regression analysis of the independent prognostic value of the risk score. Univariate **(A)** and multivariate **(B)** Cox regression analysis of risk score, age, clinical stage, tumor differentiation, and depth of invasion. **(C)** The AUC of risk score, age, clinical stage, tumor differentiation, and depth of invasion was calculated from the total survival risk score according to the ROC curve. **(D)** Relationship between lncRNA expression and age, clinical stage, tumor differentiation, and depth of invasion. **(E)** Prognostic value of PRRT3-AS, LINC01503, CDKN2B-AS, LINC0162, LINC0183, and LINC01936 in UCEC analyzed using Kaplan–Meier (KM) Plotter. *P < 0.05, **P < 0.01, ***P < 0.001.

Next, we investigated whether the prognosis of the six immune-related lncRNAs was related to clinicopathological factors, such as age, clinical stage, tumor differentiation, and depth of invasion. Univariate Cox regression analysis showed that age, depth of tumor invasion, and risk score correlated significantly with OS in patients with EC (Figure 4A), while multivariate Cox regression analysis showed that the risk score significantly correlated with OS (Figure 4B), suggesting that these six lncRNAs act as independent prognostic factors in patients with EC. To determine the sensitivity and specificity of the risk score for evaluating the prognosis of patients with EC, we performed time-dependent ROC analysis. The AUC of the risk score was 0.712 (Figure 4C), indicating that the six lncRNAs are highly reliable prognostic markers for EC. In addition, we produced a box plot showing the relationship between the expression levels of the lncRNAs and age, clinical stage, tumor differentiation, and depth of invasion (Figure 4D), while the KM-Plotter showed that PRRT3-AS1, LINC01503, CDKN2B-AS1, LINC01629, LINC01833, and LINC01936 significantly correlated with shorter OS in UCEC (Figure 4E). Together, these results indicate that the expression of the six immune-related lncRNAs is an independent prognostic factor for patients with EC.

### Correlation Between Long Non-coding RNA Expression and the Immune Microenvironment

**FIGURE 5:**
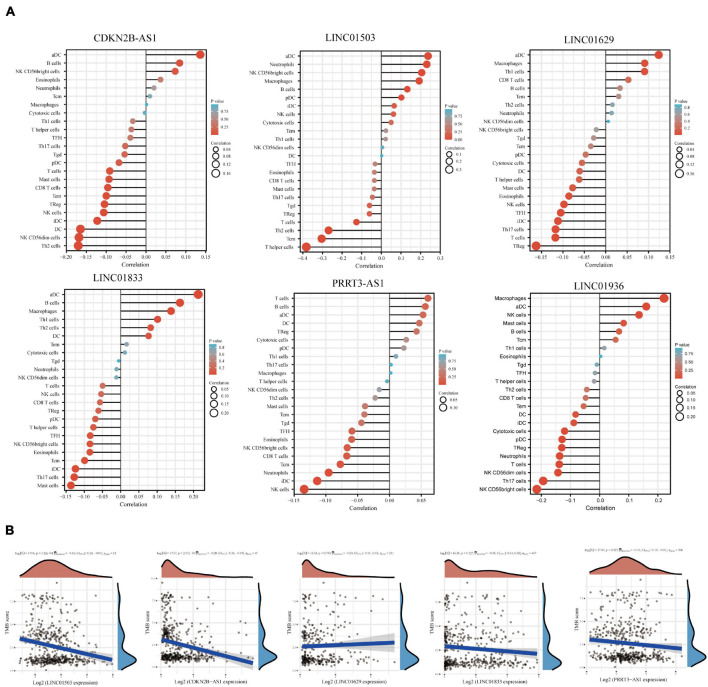
Correlation between lncRNA expression and immune infiltration. **(A)** Correlation analysis of immune-related lncRNA expression and the infiltration of aDCs, B cells, CD8+ T cells, cytotoxic cells, DCs, eosinophils, iDCs, macrophages, mast cells, neutrophils, NK CD56bright cells, NK CD56dim cells, NK cells, pDCs, T cells, T helper cells, Tcm, Tem, Tfh, Tgd, Th1 cells, Th17 cells, Th2 cells, and Tregs. **(B)** Spearman correlation analysis of TMB/MSI and immune-related lncRNA expression. Horizontal axis represents gene expression distribution. Ordinate represents TMB/MSI score distribution. The density curve on the right represents the TMB/MSI score distribution trend. The upper density curve represents the gene expression distribution trend. The uppermost value represents the correlation *p*-value, correlation coefficient, and correlation calculation method. *p*-Values of <0.05 were considered significant. The correlation coefficient range was (–1, 1), with a negative number representing a negative correlation between the expression of two genes and a positive value representing a positive correlation. The closer to 1 or –1, the stronger the correlation. The closer to 0, the weaker the correlation.

To evaluate the association between the expression of the six lncRNAs and immune cell infiltration, we used the ImmLnc database to detect their correlation with immune cell types, including CD8+ T cells, DCs, neutrophils, B cells, macrophages, and CD4+ T cells. We found that CDKN2B-AS1 expression correlated negatively with CD8+ T cells and positively with B cells, macrophages, and CD4+ T cells. Conversely, LINC01503 expression correlated negatively with CD8+ T cells and macrophages but positively with neutrophils and CD4+ T cells. LINC01936 expression correlated negatively with CD8+ T cells and DCs, whereas PRRT3-AS1 expression correlated negatively with CD8+ T cells, DCs, and neutrophils. Furthermore, LINC01629 expression correlated negatively with macrophage and positively with neutrophils, while LINC01833 expression correlated negatively with CD8+ T cells and macrophages but positively with CD4+ T cells and neutrophils (Supplementary Table 2). We then used ssGSEA to analyze the correlation between immune cell infiltration and the expression of PRRT3-AS1, LINC01503, CDKN2B-AS1, LINC01629, LINC01833, and LINC01936, respectively. The results revealed that all six lncRNAs were associated with immune cell infiltration, and that the infiltration of various immune cells was related (Figure 5A and Supplementary Table 3).

Tumor mutational burden (TMB) has attracted considerable attention in immunotherapy; PD-L1 is a biomarker for predicting the response of two important PD-1 antibody treatments. Therefore, we investigated the association between TMB and the expression of PRRT3-AS1, LINC01503, CDKN2B-AS1, LINC01629, and LINC01833 using the online database assistant for clinical bioinformatics.^3^ The relationships between satellite instability and LINC01936 data were unavailable. Notably, we found a significant negative correlation between PRRT3-AS1, LINC01503, and CDKN2B-AS1 expression and TMB (Figure 5B).

**FIGURE 6:**
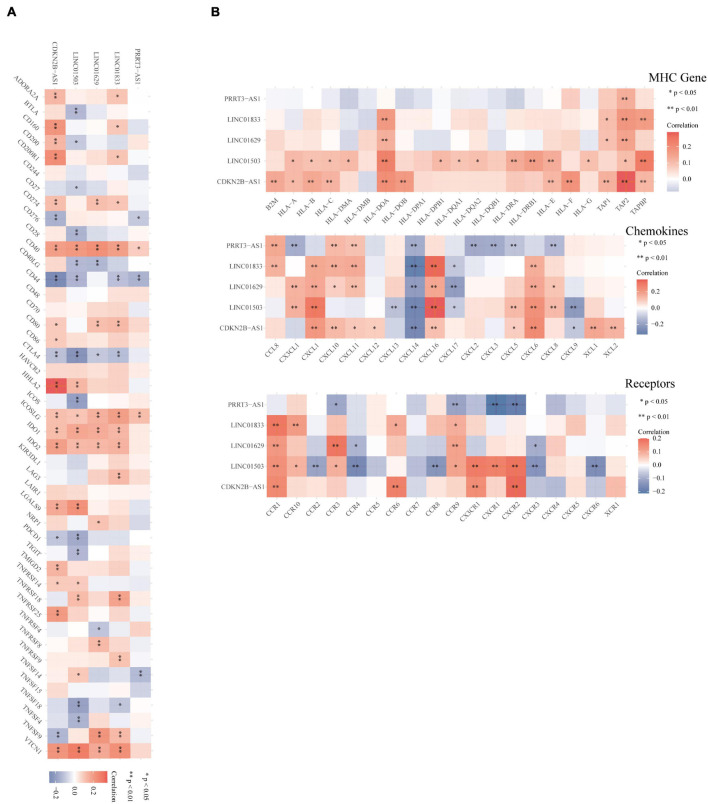
Correlation between the immune-related lncRNA expression and immune factors. **(A,B)** Spearman’s correlation analysis between immune-related lncRNAs and immune checkpoints, MHC molecules, chemokines, and chemokine receptors. Different colors represent the correlation coefficient (red, positive; blue, negative). A darker color indicates a stronger correlation, ^∗^*p* < 0.05, ^∗∗^*p* < 0.01.

In addition, we used Spearman’s correlation analysis to evaluate the relationship between PRRT3-AS1, LINC01503, CDKN2B-AS1, LINC01629, and LINC01833 and common immune checkpoints, MHC molecules, chemokines, and chemokine receptors *via* the online database assistant for clinical bioinformatics (see text footnote 3), this website was not predicted the information of LINC01936 (Figures 6A,B). All these lncRNAs displayed significant correlations with multiple immune checkpoints and immune-related genes.

### Specimen Verification

**FIGURE 7:**
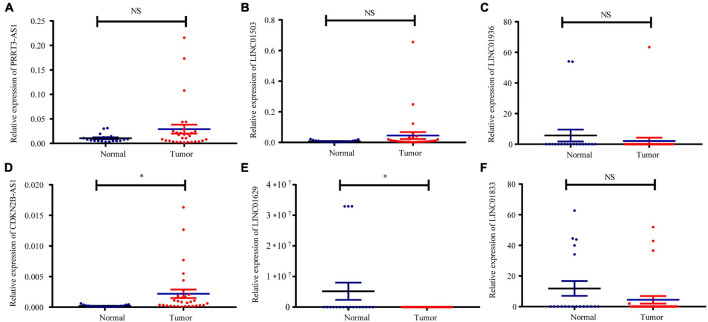
Relative expression of immune-related lncRNAs in normal and UCEC tissues. Expression of **(A)** PRRT3-AS1, **(B)** LINC01503, **(C)** LINC01936, **(D)** CDKN2B-AS1, **(E)** LINC01629, and **(F)** LINC01833 in 30 EC tissues and 19 normal tissues determined using qRT-PCR. ^∗^*p* < 0.05, NS, no statistical difference.

Fluorescent RT-qPCR was used to detect the mRNA expression of PRRT3-AS1, LINC01503, LINC01936, CDKN2B-AS1, LINC01629, and LINC01833 in 30 EC tissues and 19 normal endometrial tissues. These analyses confirmed that CDKN2B-AS1 expression is higher in EC tissues that in normal endometrial tissues (Figures 7A–F).

### CDKN2B-AS1 Knockdown Inhibits Endometrial Cancer Progression *in vitro* and *in vivo*

**FIGURE 8:**
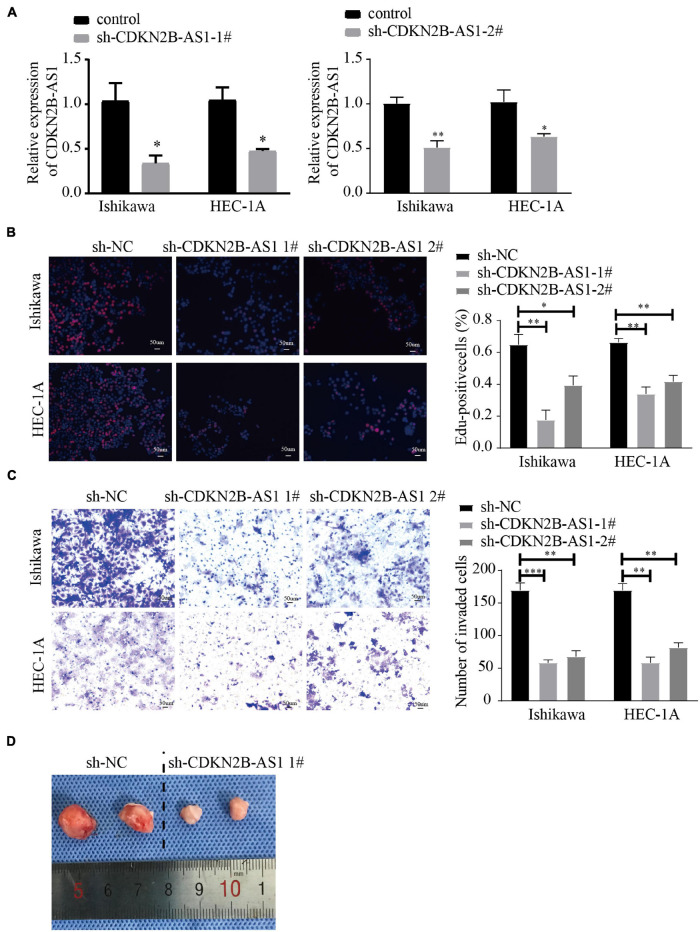
CDKN2B-AS1 knockdown inhibits malignant biological behavior in EC cells. **(A)** CDKN2B-AS1 expression in Ishikawa and HEC-1A cell lines measured using RT-qPCR. **(B)** Effect of CDKN2B-AS1 expression on proliferation determined using EdU assays in Ishikawa and HEC-1A cells. **(C)** Transwell assays to determine the number of invading cells. **(D)** Nude mice bearing tumors. A specimen from each respective group is shown (*n* = 2 per group), ^∗^*p* < 0.05, ^∗∗^*p* < 0.01, ^∗∗∗^*p* < 0.001 vs. the NC group.

Endometrial cancer is divided into two types, of which type I is estrogen-dependent, while type II is non-estrogen-dependent. The Ishikawa cell line is derived from type I tumors, and HEC-1A cells derived from type II tumors (Albitar et al., 2007). Next, we evaluated the effect of knockdown CDKN2B-AS1 on the malignant biological behavior of EC cell lines by transfecting Ishikawa and HEC-1A cells with sh-CDKN2B-AS1 chronic virus vector and a corresponding NC. We found that two construct, LV- CDKN2B-AS1 -1, LV- CDKN2B-AS1 -2 decreased the CDKN2B-AS1 expression. After the transfection efficiency had been confirmed using quantitative real-time-polymerase chain reaction (qRT-PCR; Figure 8A), EdU assays revealed that knockdown of CDKN2B-AS1 inhibited the proliferation of Ishikawa and HEC-1A cells (Figure 8B), while Transwell assays demonstrated that knockdown of CDKN2B-AS1 also inhibited their invasion ability (Figure 8C). Next, we investigated the function of CDKN2B-AS1 in an *in vivo* tumor model using nude mice (*n* = 2 per group) and found that the tumor volume was smaller in the sh-CDKN2B-AS1 than in the control groups (Figure 8D).

### CDKN2B-AS1 Cellular Localization and Functional Enrichment Analysis

**FIGURE 9:**
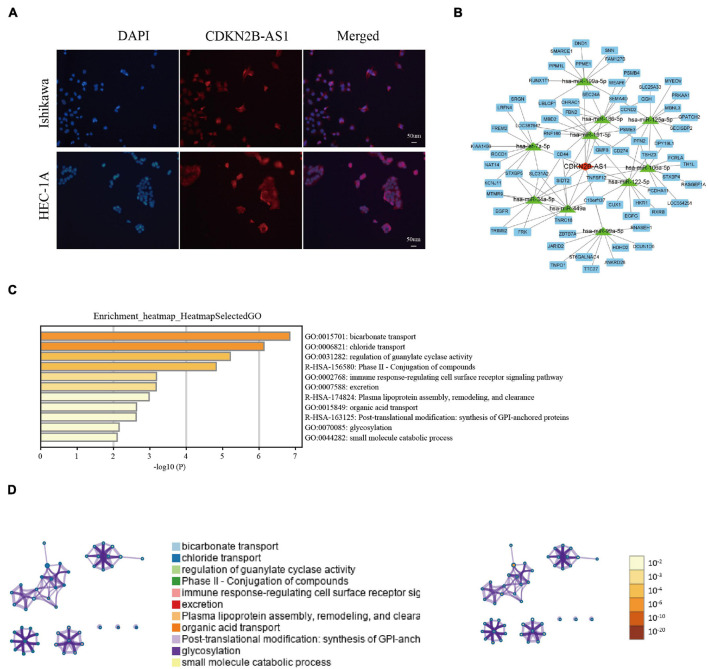
CDKN2B-AS1 cellular location, lncRNA–miRNA–mRNA regulatory network, and GSEA results. **(A)** FISH assay of CDKN2B-AS1 localization in Ishikawa and HEC-1A cells. **(B)** CDKN2B-AS1-binding miRNAs were predicted using STARBASE and their targets were retrieved from miRTarBase. The regulatory network of lncRNA-miRNA-mRNAs was visualized using Cytoscape 3.7.1. **(C,D)** Biological processes related to CDKN2B-AS1.

Since cellular localization significantly influences lncRNAs function and molecular mechanism in tumors, we investigated the subcellular localization of CDKN2B-AS1 using FISH assays. CDKN2B-AS1 was located in both the nucleus and cytoplasm of Ishikawa and HEC-1A cells (Figure 9A), suggesting that CDKN2B-AS1 exerts its biological functions *via* a ceRNA mechanism. Therefore, we analyzed the lncRNA–miRNA–mRNA network using STARBASE and miRTarBase and found that CDKN2B-AS1 was related to 10 miRNAs and 100 target genes (Figure 9B).

Considering the important role of CDKN2B-AS1 in regulating the malignant phenotype of EC cells along with our findings, we investigated the role of CDKN2B-AS1 dysregulation in the pathogenesis of EC by examining the enriched gene sets in samples with different CDKN2B-AS1 mRNA expression levels. First, we used Metascape to perform Gene Ontology (GO) enrichment analysis on the function of CDKN2B-AS1 and its top 100 related molecules (Supplementary Table 4), which were primarily involved in bicarbonate transport, chloride transport, the regulation of guanylate cyclase activity, the phase II-conjugation of compounds, immune response-regulating cell surface receptor signaling pathway, excretion plasma lipoprotein assembly, remodeling and clearance, organic acid transport, post-translational modification, synthesis of GPI-anchored proteins, glycosylation, and small molecule catabolic processes (Figures 9C,D and Supplementary Table 5). Together, these findings provide important clues regarding the mechanism underlying the pathogenesis of EC. Based on the TCGA dataset, the relationship between CDKN2B-AS1 expression and risk coefficient HR and confidence interval of clinical characteristics was analyzed by univariate Cox, and spearman correlation analysis heat map of immune score and CDKN2B-AS1 expression in multiple tumor tissues. The results suggest that CDKN2B-AS1 is an independent prognostic factor in a variety of tumors (Supplementary Figure 3A). Immune score evaluation analysis showed that the expression of CDKN2B-AS1 was related to a variety of immune cell infiltration (Supplementary Figure 3B).

## Discussion

The incidence of EC, the most common and deadly cancer among females worldwide, has been increasing worldwide. EC has a high degree of heterogeneity not only in the genes and phenotypes of EC cells, but also in the TME (Engerud et al., 2020), which contains various immune cells. Tumor and immune cells interact and form complexes. LncRNAs influence the occurrence, development, invasion, and metastasis of EC *via* immune pathways (Gao et al., 2021). Therefore, we explored immune-related lncRNAs in EC to improve our understanding of the mechanisms underlying EC occurrence and development, and to develop new diagnosis and therapeutic strategies, for better treatment outcomes.

To this end, we screened differentially expressed immune-related lncRNAs using TCGA database and the ImmLnc website, identifying six immune-related lncRNAs as prognostic markers: PRRT3-AS1, LINC01503, LINC01936, CDKN2B-AS1, LINC01629, and LINC01833. Importantly, the risk scores calculated using the coefficients of these six lncRNAs showed that the OS of high-risk patients with EC was significantly shorter than that of patients in the low-risk group in both the training and test data sets. Multi-factor Cox regression analysis of age, clinical stage, tumor differentiation, and depth of invasion confirmed that the six immune-related lncRNAs are an independent prognostic factor in EC. It has been shown that PRRT3-AS1 silencing in PC cells can inhibit their proliferation by activating the PPARγ gene, thereby blocking the mTOR signaling pathway (Fan et al., 2020). mTOR is an important eukaryotic cell signal whose stability affects the expression of cytokines in T cells and immunosuppression (Dumas et al., 2020). Recent research has also identified PRRT3-AS1 as an immune-related prognostic lncRNA in patients with HCC (Kong et al., 2020). Together, these studies suggest that PRRT3-AS1 may be a potential therapeutic target for immunotherapy in EC.

CDKN2B-AS1 is a multifunctional lncRNA that exerts carcinogenic effects in various tumors. In renal clear cell carcinoma, CDKN2B-AS1 exerts its carcinogenic activity by recruiting CREB-binding protein and three epigenetic modification complexes containing SET and MYND domains to the promoter region of the Ndc80 mitochondrial complex (NUF2) (Xie et al., 2021). In lung cancer, CDKN2B-AS1 acts as a cavernous body by adsorbing miR-378b and regulating miR-378b/NR2C2 to promote cancer development (Wang et al., 2020). However, the expression and role of CDKN2B-AS1 in EC remains unclear. LINC01503 acts as a carcinogen in cancers such as lung cancer (Zhang et al., 2020), bowel cancer (Wei et al., 2020), and gastric cancer (Ma et al., 2021), and promotes tumor progression. Previous analyses of TCGA database have shown that the lncRNA LINC01833 is associated with glycolysis and that its high expression is correlated to poor prognosis in patients with EC (Jiang et al., 2021); however, its expression and role in EC warrant further study.

To investigate the association between these immune-related lncRNAs and immune cell infiltration, we used the ImmLnc database to analyze the correlation between PRRT3-AS1, LINC01503, CDKN2B-AS1, LINC01629, and LINC01833 and CD8+ T cells, DCs, neutrophils, B cells, macrophages, and CD4+ T cells. We found that all six lncRNAs were associated with the infiltration of various interrelated immune cells. Considerable evidence suggests that CD8+ T cell subsets play important roles in tumor control, as reflected by the relationship between the number of CD8+ T cells in the tumor before treatment and the response to PD-1 therapy (Kim et al., 2021). After circulating CD8+ T cells infiltrate tumor tissues, they are activated by tumor antigens to transform into effector CD8+ T cells which kill tumor cells. In addition, CD4+ T helper cells help DCs to prepare and activate CD8+ T cells (Yang et al., 2020). Meanwhile, CD4+ Treg cell-mediated anti-tumor immunosuppression is the main mechanism of tumor immune evasion and immunotherapy resistance (Polanczyk et al., 2019). Effector CD8+ T cells gradually degenerate due to continuous tumor antigen stimulation, immunosuppressive cell suppression (e.g., Treg cells and immunosuppressive B cells), and imbalance between physical and chemical status, thereby reducing their proliferation and secretion of effector cytokines (IL-6, TNF-α, and IFN-γ), known as “T cell exhaustion” (Chen et al., 2021). Thus, the repair of CD8+ T cell anti-cancer immune activity has become the greatest limitation in tumor immunotherapy. T cell dysfunction in human tumors is characterized by an increase in the expression of inhibitory receptors (e.g., PD-1, LAG3, TIM3, 2B4, CD200, and CTLA4) on the cell surface; therefore, immune checkpoint inhibitors can partially reverse T cell depletion (Li et al., 2021).

We also analyzed the correlation between the lncRNAs and common immune checkpoints using Spearman’s correlation analysis. Notably, LINC01629, LINC01833, and CDKN2B-AS1 correlated positively with the immune checkpoint CD274, while LINC01503, CDKN2B-AS1, LINC01629, and LINC01833 correlated positively with IDO1 and IDO2. IDO1 and PD-L1 have become important targets for tumor immunotherapy and multiple inhibitors have entered clinical trials and achieved certain efficacy (Takada et al., 2020). Therefore, our findings provide new theoretical targets for EC immunotherapy.

Tumor cells display altered MHC antigen expression on their surface. The weakening of MHC antigen expression reduces the functional presentation of antigens to immune cells and thus the activation of helper T cells (Algarra et al., 2021). However, some tumors can also escape the lysis caused by cytotoxic T lymphocytes and NK cells by overexpressing non-classical MHC-I molecules, thereby escaping immune system surveillance (Catalán et al., 2015). When we analyzed the correlation between PRRT3-AS1, LINC01503, LINC01936, CDKN2B-AS1, LINC01629, and LINC01833 and MHC molecules, we found that all six immune-related lncRNAs correlated positively with multiple MHC-related molecules.

In the TME, various chemokines can be secreted by tumor, immune, and stromal cells, which ultimately activate multiple signal pathways *via* cell surface receptors and recruit immune cell subgroups to the TME, thereby spatiotemporally regulating the tumor immune response. The chemokine network can either promote or inhibit tumor cell growth, invasion, and metastasis by influencing tumor cells or tumor-related immune cells (Valeta-Magara et al., 2019). Thus, targeting chemokines and their receptors is an effective treatment strategy against malignant tumors. The findings of our Spearman’s analysis of the correlation between the immune-related lncRNAs and chemokines and their receptors will therefore help to discover new approaches for efficient and specific EC treatments based on targeted multi-chemokines.

Unfortunately, the efficacy of PD-1/PD-L1 blockade therapy in most tumors is approximately 40%; therefore, it is important to accurately screen patients who can benefit from the treatment to avoid treatment-related adverse reactions (Yin et al., 2021). One biomarker for screening patients who may benefit from PD-1/PD-L1 blockade therapy is TMB, which represents the number of mutations per megabase in the coding region of gene exons in a tumor sample. A higher TMB elicits stronger antigenicity, enabling more immune T cells to recognize and kill tumor cells in the TME (Li et al., 2020). EC is divided into four molecular types namely POLE (7%), MSI (28%), CNL (39%), and CNH (26%). EC has the highest known POLE mutation rate, meaning that POLE mutant EC has a very high mutational load. Indeed, POLE and MSI hypermutation type EC have by far the highest TMBs of all four molecular types (232 × 106 and 18 × 106/Mb vs. 2.6 × 106 and 2.9 × 106/Mb), suggesting that patients with POLE and MSI hypermutation EC are most likely to benefit from PD-1/PD-L1 blockade therapy (Gargiulo et al., 2016). In this study, we analyzed the correlation between PRRT3-AS1, LINC01503, CDKN2B-AS1, LINC01629, LINC01833, and TMB, finding that the expression of PRRT3-AS1, LINC01503, CDKN2B-AS1, and LINC01833 correlated significantly and negatively with the distribution of TMB.

To verify the findings of our bioinformatics analyses, we detected the expression of PRRT3-AS1, LINC01503, LINC01936, CDKN2B-AS1, LINC01629, and LINC01833 in EC and normal endometrial tissues using RT-qPCR. Interestingly, CDKN2B-AS1 was significantly overexpressed in EC tissues; however, its expression and function in EC remain poorly understood. We also found that CDKN2B-AS1 knockdown in Ishikawa and HEC-1A cells inhibited their proliferation and invasion. Moreover, interference with CDKN2B-AS1 *in vivo* inhibited the growth of EC cell-transplanted tumors in nude mice. FISH assays further revealed that CDKN2B-AS1 is located in the nucleus and cytoplasm of Ishikawa and HEC-1A cells. Since the subcellular location of lncRNAs influences their potential functions and underlying mechanisms in tumors, these results suggest that CDKN2B-AS1 achieves its biological function *via* a ceRNA mechanism; therefore, we constructed a ceRNA regulatory network. The microenvironment of EC is very complicated compared with that of other malignant tumors, and hence its microstructure and correlation with prognosis warrant further investigation. Therefore, future studies, with a larger sample size and *in vitro* experiments, must examine the immune regulation mechanism of EC in greater depth. In addition, RNA-Seq of EC cell lines treated with sh-CDKN2B-AS1 will help us to further study the important mechanism that CDKN2B-AS1 plays in the immune regulation and malignant progression of EC.

## Conclusion

In conclusion, this study identified six lncRNAs as a prognostic signature for EC associated with the infiltration of immune cell subtypes. Together, our results provide a new strategy for prognostic evaluation of EC, as well as possible therapeutic targets and a theoretical basis for individualized EC immunotherapy.

## Data Availability Statement

The datasets presented in this study can be found in online repositories. The names of the repository/repositories and accession number(s) can be found in the article/Supplementary Material.

## Ethics Statement

The studies involving human participants were reviewed and approved by the Ethics Committee of Shengjing Hospital Affiliated to China Medical University (Ethics number: 2018PS251K). Written informed consent for participation was not required for this study in accordance with the national legislation and the institutional requirements. The animal study was reviewed and approved by the Scientific Research and New Technology Ethical Committee of the Shengjing Hospital of China Medical University (Ethics number: 2018PS251K).

## Author Contributions

DY performed most of the experiments and contributed toward writing the manuscript. DY and X-XM conceived the study, participated in its design and coordination, and helped draft the manuscript. DY and JM performed qRT-PCR and cell culture experiments. All authors read and approved the final manuscript.

## Conflict of Interest

The authors declare that the research was conducted in the absence of any commercial or financial relationships that could be construed as a potential conflict of interest.

## Publisher’s Note

All claims expressed in this article are solely those of the authors and do not necessarily represent those of their affiliated organizations, or those of the publisher, the editors and the reviewers. Any product that may be evaluated in this article, or claim that may be made by its manufacturer, is not guaranteed or endorsed by the publisher.

## Footnotes

1
http://bio-bigdata.hrbmu.edu.cn/ImmLnc

2
http://metascape.org

3
https://www.aclbi.com/static/index.html#/
