# Supplementary material for: CDKN2B-AS1 Promotes Malignancy as a Novel Prognosis-Related Molecular Marker in the Endometrial Cancer Immune Microenvironment
Source: Front Cell Dev Biol. 2021 Oct 12;9:721676. doi: 10.3389/fcell.2021.721676 (PMC8546264; doi:10.3389/fcell.2021.721676)
Supplement: Supplementary file 1 [file Data_Sheet_1.PDF]

Supplementary Table S1: Primer sequences for qRT-PCR and sh-RNA sequences.

Name	Sequence
CDKN2B-AS1	F: GCCGCTCCGCTCCTCTTCTAG
	R: CGTGTCCAGATGTCGCGTCAG
PRRT3-AS1	F: ACCTGCCTCATTGCGTTGTGAAG
	R: ACCAGCCTGGATGACAGAGTGAG
LINC01629	F: GGCTCTAGGAATGCAGTGCAAGAC
	R: CTCTCTGTGGGCTTGTCAAGAACC
LINC01503	F: GATGTGTGACTGCTGCCAGGAG
	R: GTGGGGAGGGTGTATTCAGAGAGG
LINC01833	F: TGTTGGGTTTGGGAGGCTGTTATG
	R: GTGTAGAGGTGAGCTGGCGAATAC
LINC01936	F: GCAAGAGCCGGTAAACCCAGAAG
	R: GCCACCACAGTGAGTGTTGAGAG
GAPDH	F: GCACCGTCAAGGCTGAGAAC
	R: TGGTGAAGACGCCAGTGGA
sh-CDKN2B-AS1-1	F: CCACAUCCCUUGGAGUAAUTT
	AUUACUCCAAGGGAUGUGGTT
sh-CDKN2B-AS1-2	F: GCAAUAGCAUGUCAAACAATT
	UUGUUUGACAUGCUAUUGCTT

Supplementary Table S2: Correlation of 6 lncRNAs with immune infiltration analyzed by ImmucLnc.

IncRNA ID	IncRNA Symbol	Immune Cell	P Value	Rs Value
ENSG00000240498	CDKN2B-AS1	CD8_Tcell	0	-0.204
ENSG00000240498	CDKN2B-AS1	Dendritic	0.263	-0.048
ENSG00000240498	CDKN2B-AS1	Neutrophil	0.126	0.066
ENSG00000240498	CDKN2B-AS1	B_cell	0.004	0.124
ENSG00000240498	CDKN2B-AS1	Macrophage	0.003	0.127
ENSG00000240498	CDKN2B-AS1	CD4_Tcell	0	0.158
ENSG00000233901	LINC01503	CD8_Tcell	0	-0.314
ENSG00000233901	LINC01503	Macrophage	0.013	-0.107
ENSG00000233901	LINC01503	Dendritic	0.922	-0.004
ENSG00000233901	LINC01503	B_cell	0.469	0.031
ENSG00000233901	LINC01503	Neutrophil	0.002	0.13
ENSG00000233901	LINC01503	CD4_Tcell	0.001	0.141
ENSG00000235997	LINC01936	CD8_Tcell	0	-0.238
ENSG00000235997	LINC01936	Dendritic	0.005	-0.12
ENSG00000235997	LINC01936	Macrophage	0.693	-0.017
ENSG00000235997	LINC01936	B_cell	0.74	-0.014
ENSG00000235997	LINC01936	Neutrophil	0.271	0.047
ENSG00000235997	LINC01936	CD4_Tcell	0.114	0.068
ENSG00000230082	PRRT3-AS1	CD8_Tcell	0	-0.183
ENSG00000230082	PRRT3-AS1	Dendritic	0	-0.172
ENSG00000230082	PRRT3-AS1	Neutrophil	0.006	-0.119
ENSG00000230082	PRRT3-AS1	Macrophage	0.667	-0.018
ENSG00000230082	PRRT3-AS1	B_cell	0.87	0.007
ENSG00000230082	PRRT3-AS1	CD4_Tcell	0.799	0.011
ENSG00000258602	LINC01629	Macrophage	0	-0.176
ENSG00000258602	LINC01629	B_cell	0.24	-0.05
ENSG00000258602	LINC01629	CD8_Tcell	0.499	-0.029
ENSG00000258602	LINC01629	CD4_Tcell	0.383	0.037
ENSG00000258602	LINC01629	Dendritic	0.253	0.049
ENSG00000258602	LINC01629	Neutrophil	0	0.182
ENSG00000259439	LINC01833	CD8_Tcell	0	-0.159
ENSG00000259439	LINC01833	Macrophage	0.01	-0.11
ENSG00000259439	LINC01833	Dendritic	0.981	-0.001
ENSG00000259439	LINC01833	B_cell	0.404	0.036
ENSG00000259439	LINC01833	CD4_Tcell	0.013	0.106
ENSG00000259439	LINC01833	Neutrophil	0.001	0.138

Supplementary Table S3: Correlation of 6 lncRNAs with immune infiltration analyzed by ssGSEA.

lmcRNA	Immune Cells	Correlation coefficient (Pearson)	P(Pearson)	Correlation coefficient(Spearman)	P(Spearman)
CDKN2B-AS1	aDC	0.058	0.175	0.136	0.001
CDKN2B-AS1	B cells	0.002	0.969	0.084	0.047
CDKN2B-AS1	CD8 T cells	-0.284	<0.001	-0.096	0.024
CDKN2B-AS1	Cytotoxic cells	-0.123	0.004	-0.003	0.936
CDKN2B-AS1	DC	-0.238	<0.001	-0.165	<0.001
CDKN2B-AS1	Eosinophils	-0.063	0.139	0.036	0.392
CDKN2B-AS1	iDC	-0.271	<0.001	-0.122	0.004
CDKN2B-AS1	Macrophages	-0.007	0.863	0.001	0.974
CDKN2B-AS1	Mast cells	-0.165	<0.001	-0.093	0.029
CDKN2B-AS1	Neutrophils	-0.024	0.572	0.020	0.638
CDKN2B-AS1	NK CD56bright cells	-0.111	0.009	0.073	0.086
CDKN2B-AS1	NK CD56dim cells	-0.214	<0.001	-0.168	<0.001
CDKN2B-AS1	NK cells	-0.332	<0.001	-0.106	0.013
CDKN2B-AS1	pDC	-0.175	<0.001	-0.068	0.111
CDKN2B-AS1	T cells	-0.151	<0.001	-0.091	0.032
CDKN2B-AS1	T helper cells	0.029	0.496	-0.037	0.389
CDKN2B-AS1	Tcm	0.178	<0.001	0.010	0.818
CDKN2B-AS1	Tem	-0.083	0.053	-0.100	0.019
CDKN2B-AS1	TFH	-0.137	0.001	-0.039	0.356
CDKN2B-AS1	Tgd	-0.028	0.507	-0.054	0.207
CDKN2B-AS1	Th1 cells	-0.078	0.069	-0.033	0.435
CDKN2B-AS1	Th17 cells	-0.071	0.094	-0.052	0.222
CDKN2B-AS1	Th2 cells	-0.064	0.131	-0.170	<0.001
CDKN2B-AS1	TReg	-0.203	<0.001	-0.104	0.014
LINC01503	aDC	0.222	<0.001	0.238	<0.001
LINC01503	B cells	0.103	0.015	0.132	0.002
LINC01503	CD8 T cells	-0.034	0.427	-0.035	0.405
LINC01503	Cytotoxic cells	0.035	0.407	0.050	0.243
LINC01503	DC	0.010	0.808	0.002	0.970
LINC01503	Eosinophils	-0.072	0.089	-0.035	0.406
LINC01503	iDC	0.030	0.484	0.065	0.126
LINC01503	Macrophages	0.160	<0.001	0.193	<0.001
LINC01503	Mast cells	-0.062	0.147	-0.036	0.396
LINC01503	Neutrophils	0.206	<0.001	0.231	<0.001
LINC01503	NK CD56bright cells	0.158	<0.001	0.206	<0.001
LINC01503	NK CD56dim cells	0.019	0.651	0.005	0.905
LINC01503	NK cells	0.055	0.195	0.062	0.148
LINC01503	pDC	0.113	0.008	0.102	0.016
LINC01503	T cells	-0.141	<0.001	-0.127	0.003

LINC01503	T helper cells	-0.405	<0.001	-0.383	<0.001
LINC01503	Tcm	-0.319	<0.001	-0.304	<0.001
LINC01503	Tem	0.032	0.456	0.023	0.588
LINC01503	TFH	-0.058	0.172	-0.032	0.451
LINC01503	Tgd	-0.098	0.021	-0.060	0.156
LINC01503	Th1 cells	0.016	0.712	0.022	0.609
LINC01503	Th17 cells	-0.025	0.560	-0.046	0.285
LINC01503	Th2 cells	-0.260	<0.001	-0.269	<0.001
LINC01503	TReg	-0.060	0.157	-0.061	0.154
LINC01629	aDC	0.049	0.254	0.124	0.004
LINC01629	B cells	0.043	0.318	0.033	0.435
LINC01629	CD8 T cells	0.005	0.907	0.053	0.216
LINC01629	Cytotoxic cells	-0.045	0.294	-0.055	0.193
LINC01629	DC	-0.032	0.450	-0.061	0.152
LINC01629	Eosinophils	-0.119	0.005	-0.086	0.043
LINC01629	iDC	-0.129	0.002	-0.112	0.009
LINC01629	Macrophages	0.097	0.022	0.091	0.033
LINC01629	Mast cells	-0.063	0.138	-0.077	0.070
LINC01629	Neutrophils	0.029	0.499	0.014	0.747
LINC01629	NK CD56bright cells	-0.070	0.102	-0.022	0.602
LINC01629	NK CD56dim cells	0.086	0.043	0.006	0.887
LINC01629	NK cells	-0.183	<0.001	-0.098	0.022
LINC01629	pDC	-0.026	0.541	-0.047	0.272
LINC01629	T cells	-0.099	0.020	-0.118	0.006
LINC01629	T helper cells	-0.077	0.070	-0.062	0.145
LINC01629	Tcm	0.007	0.878	-0.035	0.414
LINC01629	Tem	0.044	0.298	0.030	0.481
LINC01629	TFH	-0.104	0.014	-0.105	0.013
LINC01629	Tgd	0.021	0.630	-0.028	0.509
LINC01629	Th1 cells	0.127	0.003	0.091	0.033
LINC01629	Th17 cells	-0.116	0.006	-0.117	0.006
LINC01629	Th2 cells	0.044	0.303	0.016	0.715
LINC01629	TReg	-0.126	0.003	-0.163	<0.001
LINC01833	aDC	0.141	<0.001	0.213	<0.001
LINC01833	B cells	0.070	0.102	0.163	<0.001
LINC01833	CD8 T cells	-0.106	0.013	-0.057	0.182
LINC01833	Cytotoxic cells	-0.057	0.180	0.012	0.787
LINC01833	DC	0.030	0.486	0.077	0.072
LINC01833	Eosinophils	-0.078	0.067	-0.085	0.047
LINC01833	iDC	-0.157	<0.001	-0.124	0.003
LINC01833	Macrophages	0.120	0.005	0.138	0.001
LINC01833	Mast cells	-0.144	<0.001	-0.137	0.001
LINC01833	Neutrophils	-0.079	0.063	-0.011	0.790
LINC01833	NK CD56bright cells	-0.121	0.004	-0.084	0.048

LINC01833	NK CD56dim cells	-0.027	0.522	-0.012	0.785
LINC01833	NK cells	-0.058	0.172	-0.054	0.209
LINC01833	pDC	-0.117	0.006	-0.070	0.103
LINC01833	T cells	-0.082	0.053	-0.050	0.245
LINC01833	T helper cells	-0.072	0.089	-0.075	0.079
LINC01833	Tcm	-0.060	0.156	-0.099	0.020
LINC01833	Tem	-0.024	0.581	0.016	0.701
LINC01833	TFH	-0.125	0.003	-0.083	0.050
LINC01833	Tgd	-0.038	0.376	-0.006	0.896
LINC01833	Th1 cells	0.063	0.141	0.101	0.017
LINC01833	Th17 cells	-0.097	0.023	-0.127	0.003
LINC01833	Th2 cells	0.075	0.079	0.082	0.053
LINC01833	TReg	-0.096	0.024	-0.061	0.152
PRRT3-AS1	aDC	0.051	0.229	0.053	0.215
PRRT3-AS1	B cells	0.047	0.267	0.056	0.186
PRRT3-AS1	CD8 T cells	-0.078	0.068	-0.067	0.115
PRRT3-AS1	Cytotoxic cells	0.036	0.402	0.026	0.537
PRRT3-AS1	DC	0.048	0.257	0.047	0.271
PRRT3-AS1	Eosinophils	-0.061	0.154	-0.059	0.163
PRRT3-AS1	iDC	-0.121	0.005	-0.114	0.008
PRRT3-AS1	Macrophages	0.016	0.715	0.002	0.961
PRRT3-AS1	Mast cells	-0.038	0.375	-0.038	0.367
PRRT3-AS1	Neutrophils	-0.100	0.019	-0.096	0.025
PRRT3-AS1	NK CD56bright cells	-0.107	0.012	-0.066	0.119
PRRT3-AS1	NK CD56dim cells	-0.019	0.660	-0.016	0.705
PRRT3-AS1	NK cells	-0.134	0.002	-0.134	0.002
PRRT3-AS1	pDC	0.004	0.928	0.023	0.597
PRRT3-AS1	T cells	0.066	0.120	0.060	0.156
PRRT3-AS1	T helper cells	-0.008	0.854	-0.004	0.928
PRRT3-AS1	Tcm	-0.045	0.295	-0.077	0.069
PRRT3-AS1	Tem	-0.043	0.311	-0.039	0.355
PRRT3-AS1	TFH	-0.070	0.101	-0.059	0.166
PRRT3-AS1	Tgd	-0.018	0.677	-0.044	0.300
PRRT3-AS1	Th1 cells	0.013	0.758	0.010	0.814
PRRT3-AS1	Th17 cells	0.001	0.990	0.002	0.954
PRRT3-AS1	Th2 cells	-0.017	0.689	-0.023	0.598
PRRT3-AS1	TReg	0.045	0.293	0.043	0.318
LINC01936	aDC	0.147	<0.001	0.160	<0.001
LINC01936	B cells	0.052	0.222	0.067	0.117
LINC01936	CD8 T cells	-0.110	0.010	-0.048	0.263
LINC01936	Cytotoxic cells	-0.131	0.002	-0.120	0.005
LINC01936	DC	-0.056	0.187	-0.082	0.053
LINC01936	Eosinophils	-0.086	0.044	0.004	0.921
LINC01936	iDC	-0.147	<0.001	-0.089	0.038

LINC01936	Macrophages	0.229	<0.001	0.221	<0.001
LINC01936	Mast cells	-0.096	0.024	0.082	0.053
LINC01936	Neutrophils	-0.195	<0.001	-0.137	0.001
LINC01936	NK CD56bright cells	-0.225	<0.001	-0.215	<0.001
LINC01936	NK CD56dim cells	-0.151	<0.001	-0.143	<0.001
LINC01936	NK cells	0.039	0.359	0.135	0.001
LINC01936	pDC	-0.164	<0.001	-0.129	0.002
LINC01936	T cells	-0.153	<0.001	-0.138	0.001
LINC01936	T helper cells	-0.062	0.144	-0.019	0.663
LINC01936	Tcm	-0.022	0.600	0.055	0.197
LINC01936	Tem	-0.076	0.076	-0.055	0.200
LINC01936	TFH	-0.084	0.048	-0.016	0.712
LINC01936	Tgd	-0.048	0.263	-0.010	0.820
LINC01936	Th1 cells	0.006	0.891	0.016	0.709
LINC01936	Th17 cells	-0.097	0.022	-0.194	<0.001
LINC01936	Th2 cells	-0.048	0.265	-0.044	0.302
LINC01936	TReg	-0.119	0.005	-0.130	0.002

Supplementary Table S4: Most Similar Genes of CDKN2B-AS1.

Gene Symbol	Gene ID	PCC
TMIGD1	ENSG00000182271.12	0.86
GUCA2A	ENSG00000197273.3	0.83
ZG16	ENSG00000174992.7	0.82
BEST4	ENSG00000142959.4	0.78
CA7	ENSG00000168748.13	0.77
TMEM236	ENSG00000148483.8	0.76
NXPE4	ENSG00000137634.9	0.74
CA1	ENSG00000133742.13	0.74
SLC26A3	ENSG00000091138.12	0.74
GUCA2B	ENSG00000044012.3	0.73
RP4-811M8.1	ENSG00000279835.1	0.73
PHGR1	ENSG00000233041.8	0.73
BTNL3	ENSG00000168903.8	0.73
OTOP2	ENSG00000183034.12	0.7
RP11-35P15.1	ENSG00000270403.1	0.7
LYPD8	ENSG00000259823.5	0.7
SLC26A2	ENSG00000155850.7	0.69
TRPM6	ENSG00000119121.21	0.69
RP11-349K16.1	ENSG00000256643.1	0.68
TUBAL3	ENSG00000178462.11	0.68
UGT2B17	ENSG00000197888.2	0.67
MYO1A	ENSG00000166866.12	0.65
DHRS11	ENSG00000278535.4	0.65
NXPE1	ENSG00000095110.7	0.65
CEACAM7	ENSG00000007306.14	0.64

RP11-747D18.1	ENSG00000239205.1	0.64
GPA33	ENSG00000143167.11	0.64
RP11-542M13.2	ENSG00000269667.1	0.63
LGALS4	ENSG00000171747.8	0.62
URAD	ENSG00000183463.5	0.62
PIGZ	ENSG00000119227.7	0.61
MEP1A	ENSG00000112818.9	0.61
RP11-209E8.1	ENSG00000259237.1	0.6
AP001187.9	ENSG00000229719.3	0.6
SATB2-AS1	ENSG00000225953.2	0.6
SLC51B	ENSG00000186198.3	0.59
PDZD3	ENSG00000172367.15	0.58
LINC00483	ENSG00000167117.8	0.58
ETHE1	ENSG00000105755.7	0.57
MS4A12	ENSG00000071203.9	0.57
SELENBP1	ENSG00000143416.20	0.56
TMEM253	ENSG00000232070.8	0.56
BEST2	ENSG00000039987.6	0.55
XXbac-B476C20.9	ENSG00000225335.3	0.55
C15orf48	ENSG00000166920.10	0.54
RP11-307N16.7	ENSG00000277040.1	0.54
KRT20	ENSG00000171431.3	0.54
SLC22A18AS	ENSG00000254827.5	0.53
TDP2	ENSG00000111802.13	0.53
RP11-77K12.7	ENSG00000260092.1	0.53
LINC00675	ENSG00000263429.3	0.53
CHP2	ENSG00000166869.2	0.53
MOGAT2	ENSG00000166391.14	0.52
AQP8	ENSG00000103375.10	0.51

BTNL8	ENSG00000113303.11	0.51
RP1-278O22.1	ENSG00000224961.1	0.5
RP11-319C21.1	ENSG00000228776.2	0.49
SLAMF6P1	ENSG00000227243.3	0.49
RN7SKP127	ENSG00000222375.1	0.49
SULT1B1	ENSG00000173597.8	0.49
RP11-172C16.4	ENSG00000256674.1	0.49
EPS8L3	ENSG00000198758.10	0.48
RP11-1060J15.5	ENSG00000230519.2	0.48
REP15	ENSG00000174236.3	0.48
CES3	ENSG00000172828.12	0.47
ISX	ENSG00000175329.12	0.47
DQX1	ENSG00000144045.13	0.47
RP11-202A13.1	ENSG00000243832.1	0.47
MUC12	ENSG00000205277.9	0.47
PPP1R14D	ENSG00000166143.9	0.47
CEACAM1	ENSG00000079385.21	0.46
IGHA2	ENSG00000211890.3	0.46
TMEM54	ENSG00000121900.18	0.45
RP11-77K12.10	ENSG00000273971.1	0.45
LRRC75A	ENSG00000181350.11	0.45
HTR4	ENSG00000164270.17	0.45
CDX1	ENSG00000113722.16	0.45
ENTPD8	ENSG00000188833.9	0.45
FABP1	ENSG00000163586.9	0.44
CHST5	ENSG00000135702.14	0.44
MTMR11	ENSG00000014914.19	0.44
CTD-2385L22.2	ENSG00000279756.1	0.44
CDHR5	ENSG00000099834.18	0.43

AC019330.1	ENSG00000225421.1	0.43
RP4-659I19.1	ENSG00000232175.1	0.43
RPL15P21	ENSG00000244753.2	0.42
MYO7B	ENSG00000169994.18	0.42
ST6GALNAC1	ENSG00000070526.14	0.42
AMN	ENSG00000166126.10	0.42
RP11-396O20.2	ENSG00000254645.1	0.42
PIGR	ENSG00000162896.5	0.42
AC007182.6	ENSG00000224721.1	0.42
CLCA4	ENSG00000016602.9	0.42
CA4	ENSG00000167434.9	0.42
RP11-1100L3.4	ENSG00000258021.1	0.42
SIRT6	ENSG00000077463.14	0.41
UGT2B29P	ENSG00000250566.1	0.41
HHLA2	ENSG00000114455.13	0.41
ADTRP	ENSG00000111863.12	0.41
FCGBP	ENSG00000275395.4	0.41

Supplementary Table S5: Significantly enriched GO annotations (Biological Processes) of CDKN2B-AS1 in endometrial carcinoma in Metascape.

GO	Category	Description	Count	%	Log10(P)	Log10(q)
GO:0015701	GO Biological Processes	bicarbonate transport	5	6.25	-6.84	-2.48
GO:0006821	GO Biological Processes	chloride transport	6	7.5	-6.13	-2.12
GO:0031282	GO Biological Processes	regulation of guanylate cyclase activity	3	3.75	-5.21	-1.55
R-HSA-156580	Reactome Gene Sets	Phase II - Conjugation of compounds	5	6.25	-4.82	-1.46
GO:0002768	GO Biological Processes	immune response-regulating cell surface receptor signaling pathway	7	8.75	-3.18	-0.25
GO:0007588	GO Biological Processes	excretion	3	3.75	-3.17	-0.25
R-HSA-174824	Reactome Gene Sets	Plasma lipoprotein assembly, remodeling, and clearance	3	3.75	-2.98	-0.1
GO:0015849	GO Biological Processes	organic acid transport	5	6.25	-2.63	0
R-HSA-163125	Reactome Gene Sets	Post-translational modification: synthesis of GPI-anchored proteins	3	3.75	-2.62	0
GO:0070085	GO Biological Processes	glycosylation	4	5	-2.15	0
GO:0044282	GO Biological Processes	small molecule catabolic process	5	6.25	-2.1	0

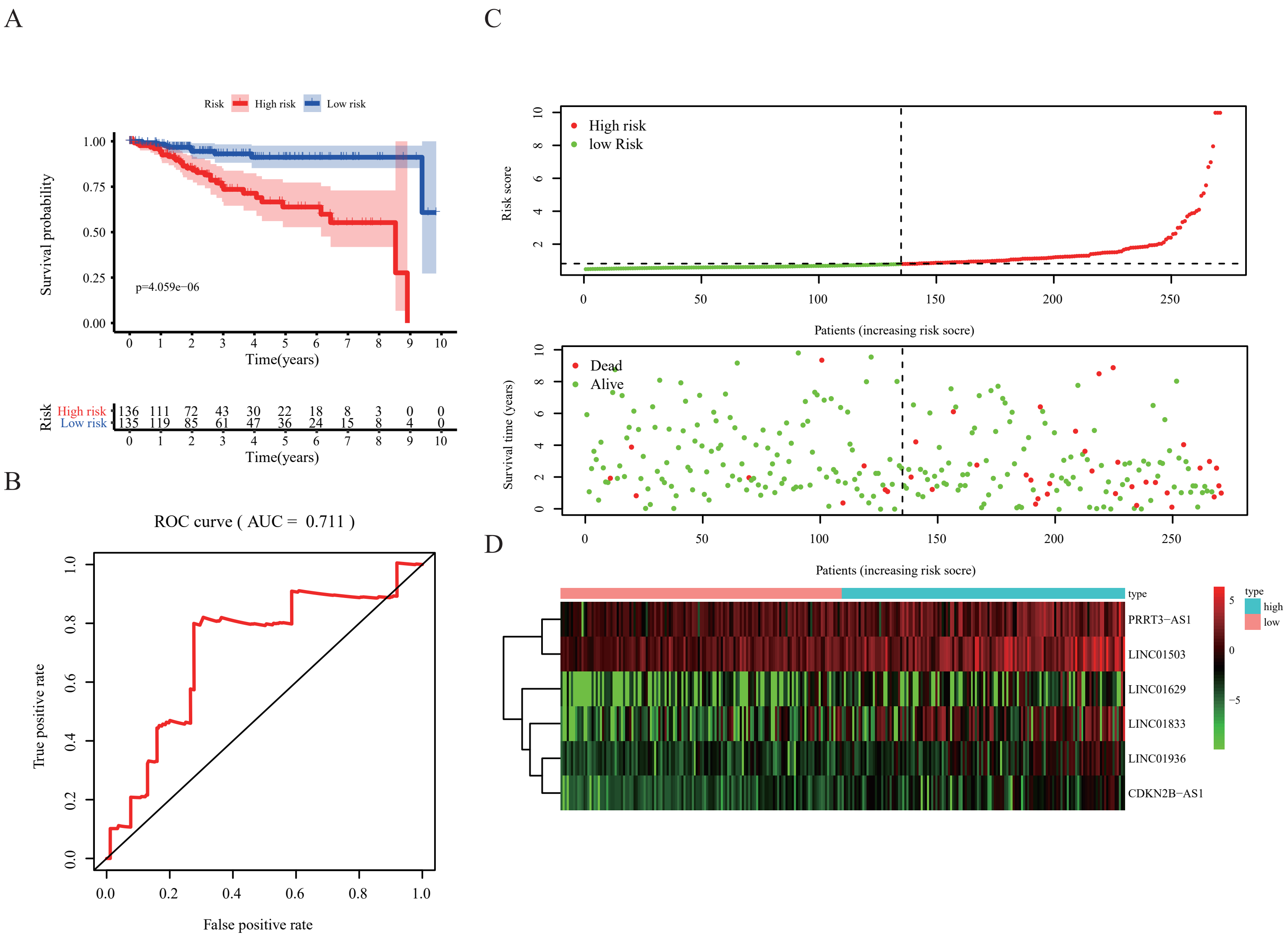

Supplementary Figure 1. Validation of the 6 immune-related lncRNAs signature for OS and the prognosis of patients with EC (Test set 1, n = 271).
A. Kaplan-Meier OS curves for the high- and low-risk groups.
B. The risk curve of each sample reordered by risk score.
C. The scatter plot of the sample survival overview. The green and red dots represent survival and death, respectively.
D. Heatmap showed the expression profiles of the signature in the low-risk groups and high-risk groups. The pink bar represented the low-risk group, and the blue bar represents the high-risk group.

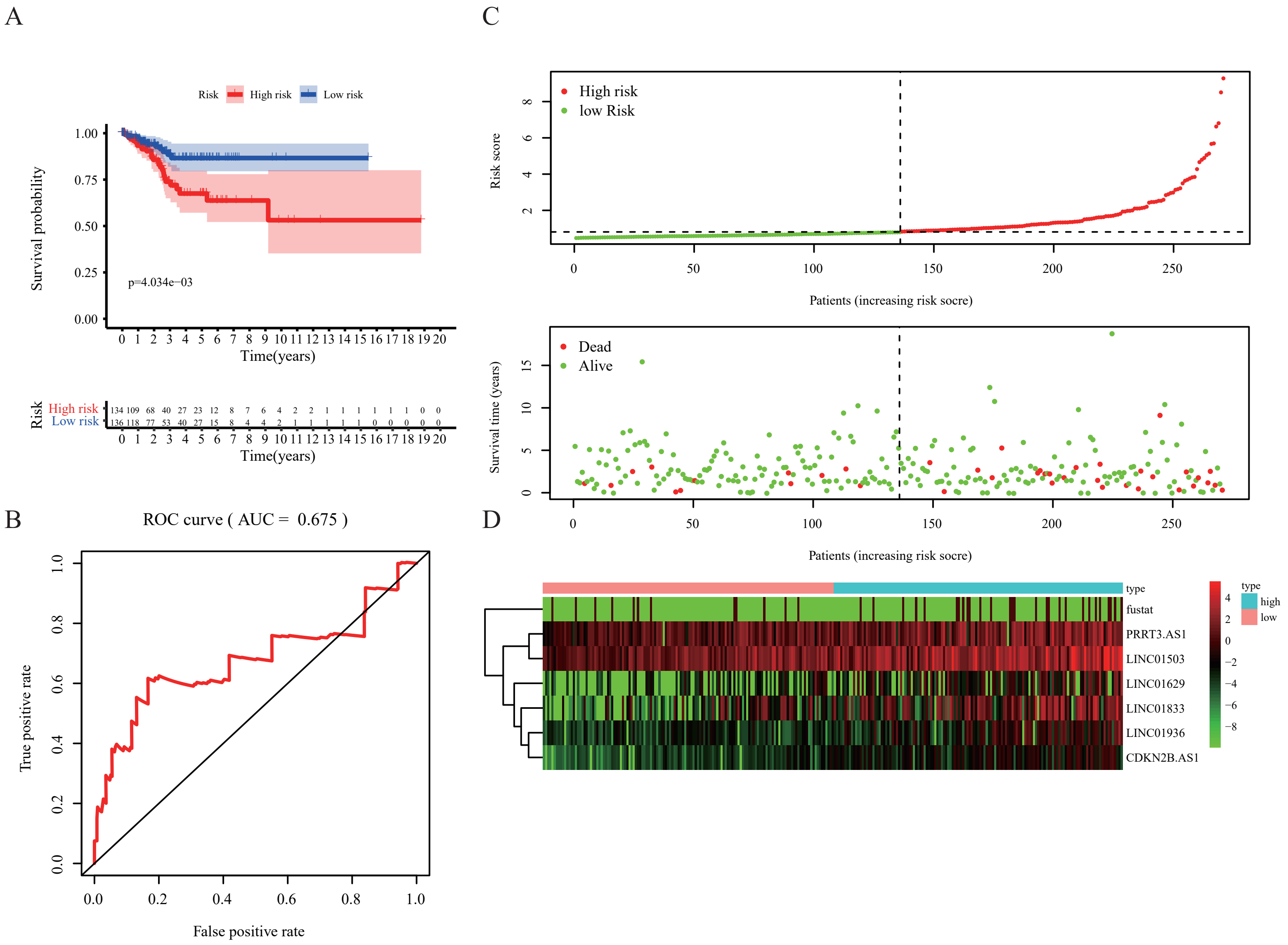

Supplementary Figure 2. Validation of the 6 immune-related lncRNAs signature for OS and the prognosis of patients with EC (Test set 2, n = 270).
A. Kaplan-Meier OS curves.
B. The risk curve.
C. The scatter plot of the sample survival overview.
D. Heatmap showed the expression profiles of the signature in the low-risk groups and high-risk groups.

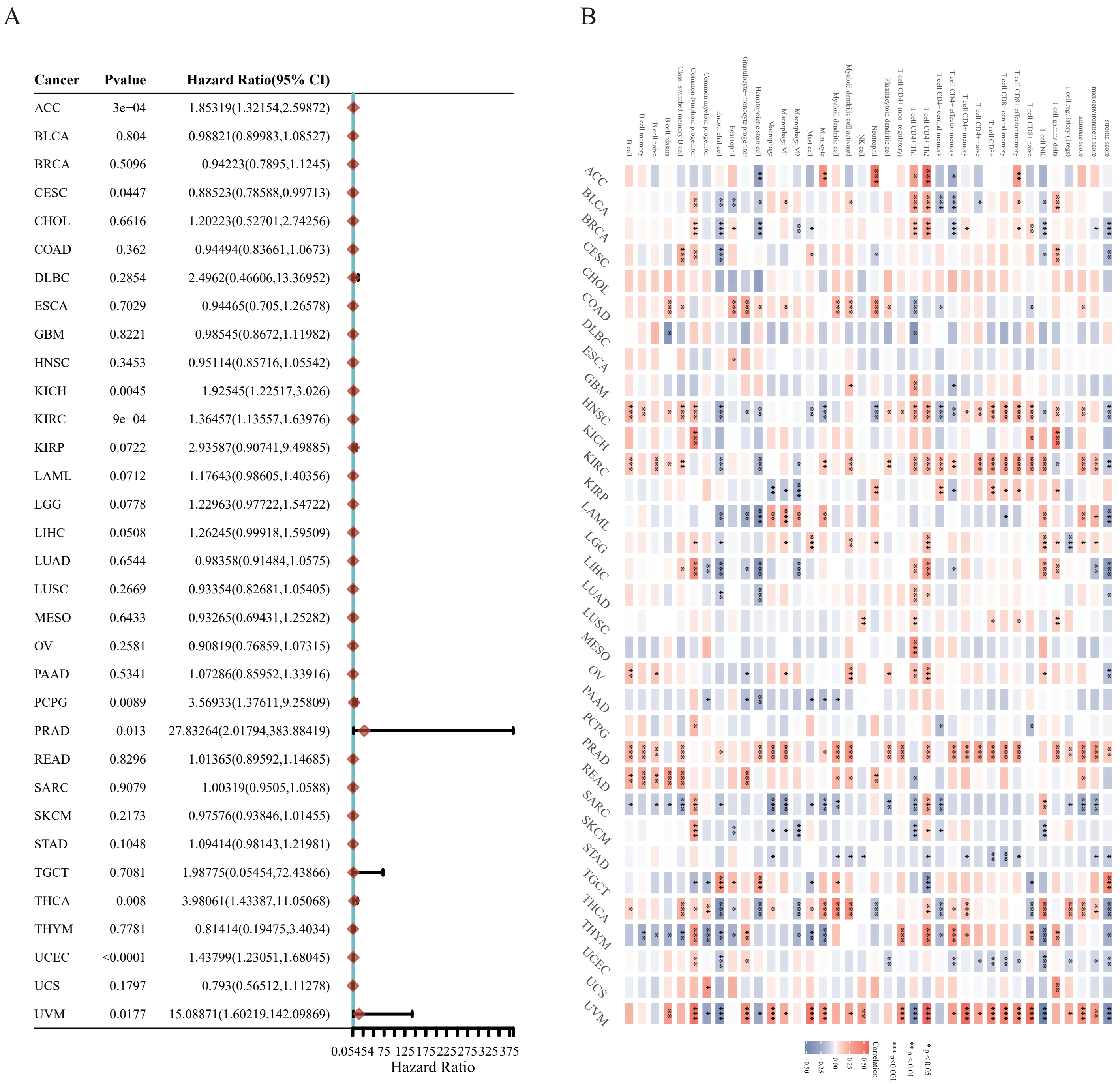

Supplementary Figure 3. Correlation between CDKN2B-AS1 expression and immune infiltration, risk coefficient.
A. Hazard ratio and p-value of constituents involved in univariate a Cox regression and some parameters of the cancers..
B. Spearman correlation analysis heat map of immune score and CDKN2B-AS1 expression in multiple tumor tissues, where the horizontal axis represents different tumor tissues, the vertical axis represents different immune scores, different colors represent correlation coefficients, and negative values represent negative correlations.
A positive value represents a positive correlation. The stronger the correlation, the darker the color, *p < 0.05, **p < 0.01, ***p < 0.001), and the asterisk represents the importance (*p).
The significance of the two groups of samples passed the Wilcox test.

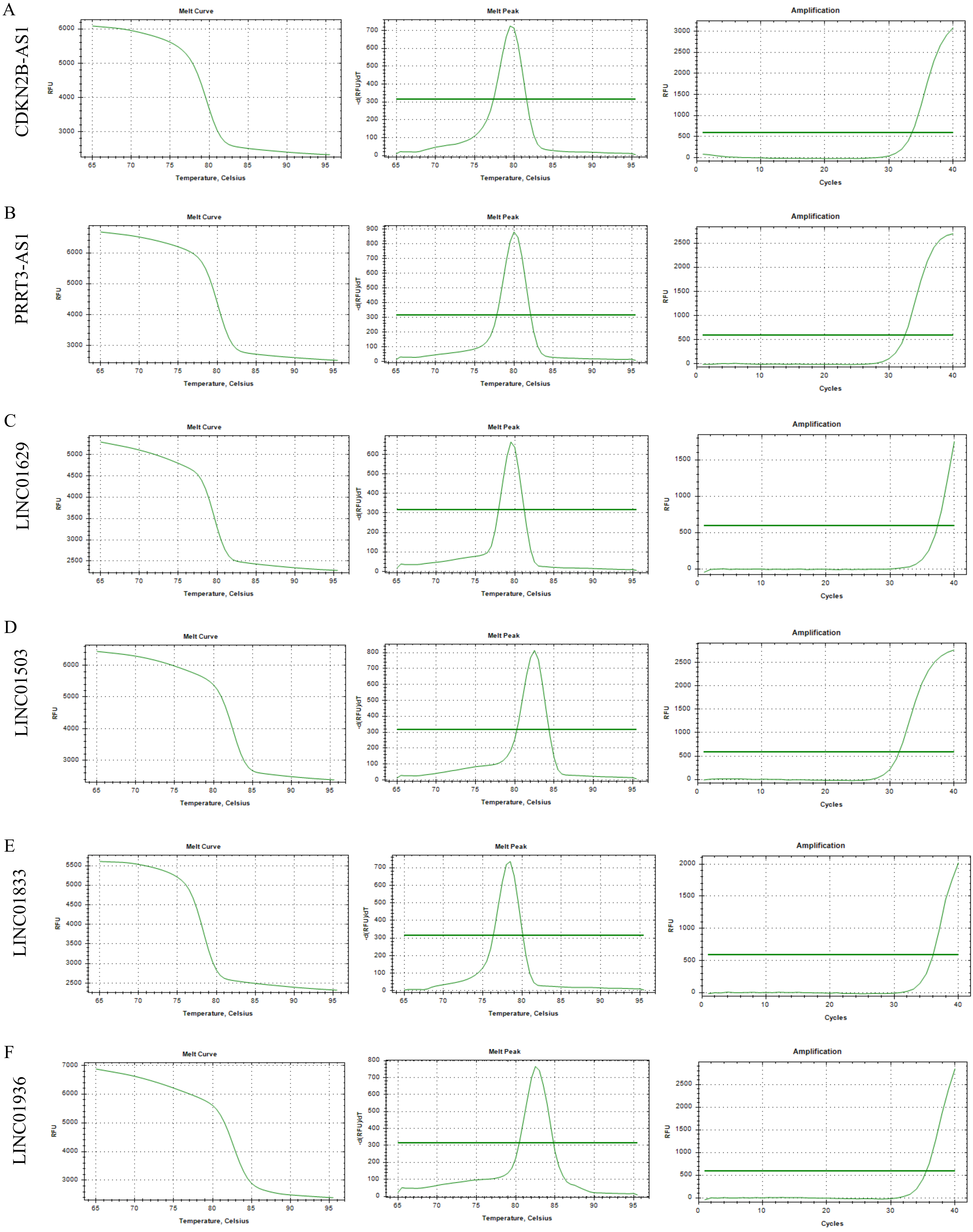

Supplementary Figure 4. Primers' amplification curve and melt curve of lncRNAs detected by Syber green qRT-PCR.
